# Building a More Resilient, Inclusive Public Health Infrastructure: Insights From Chicago's Community‐Based COVID‐19 Corps

**DOI:** 10.1002/hpm.3877

**Published:** 2024-12-01

**Authors:** Jeni Hebert‐Beirne, Sage Kim, Linda Forst, Guddi Kapadia, Alexis Grant, Alisa Velonis, Mark Dworkin, Maggie Acosta, Kim Jay, Diana Ghebenei, Caesar Thompson, Emily Stiehl

**Affiliations:** ^1^ Collaboratory for Health Justice Community Health Sciences School of Public Health (UICSPH) University of Illinois Chicago Chicago Illinois USA; ^2^ Health Policy and Administration School of Public Health University of Illinois Chicago Chicago Illinois USA; ^3^ Environmental and Occupational Health Sciences School of Public Health University of Illinois Chicago Chicago Illinois USA; ^4^ Collaboratory for Health Justice School of Public Health University of Illinois Chicago Chicago Illinois USA; ^5^ Community Health Sciences School of Public Health University of Illinois Chicago Chicago Illinois USA; ^6^ Epidemiology and Biostatistics School of Public Health University of Illinois Chicago Chicago Illinois USA; ^7^ Center for CHW Outcomes and Workforce Development (CROWD) Sinai Urban Health Institute Chicago Illinois USA; ^8^ Chicago Community Health Response Corps Latino Resource Institute Chicago Illinois USA

## Abstract

Emergency events such as natural disasters, pandemics, and other health disasters have a predictably disproportionate impact on vulnerable populations and the COVID‐19 pandemic was not an exception. To respond to potentially catastrophic consequences of COVID‐19 and to build an infrastructure for a more inclusive recovery, in June 2020, the Chicago Department of Public Health partnered with a state university school of public health, a community college that prepares students for healthcare occupations, a research institute at a private university, a public health institute affiliated with a hospital system, and a workforce development organisation. The team formed the Chicago COVID‐19 Contact Tracing Corps (ChiTracing). Centring the expertise of grassroots community‐based organisations (CBOs), ChiTracing partnered with 31 CBOs operating in the highest hardship community areas. These CBOs hired and trained over 500 community members, who had a history of unemployment, as neighbourhood‐level public health ambassadors and contact tracers, known as the ChiTracing Corps members. Informed by a shared theory of change, we brought three strategies to this work**: investing in a new public health infrastructure** by centring trusted CBOs and people with lived experience of systems of oppression as part of the public health system, **increasing awareness and knowledge** of public health and available resources for the most vulnerable, **and fostering relationships and power building** among diverse collaborators. In this paper, we highlight lessons learnt and share insights on how future efforts can bring collaborative, inclusive approaches to public health workforce development.

**Summary**

- Chicago's hyperlocal COVID‐19 response invested in CBO assets and knowledge and lived experience of Chicago residents in highest hardship neighbourhoods.
- Partner institutions collaborated to create a scaffolding of training for the ChiTracing Corps
- The Corps of community members provided outreach and resource navigation for their neighbourhoods while working on personalised career pathways.
- Networking CBOs and partner institutions while leveraging the strengths of community health workers provided insight on how to build stronger local public health systems.

## 1 Introduction

Natural disasters such as the COVID‐19 pandemic exacerbate the uneven distribution of health and economic risk and vulnerability across communities that results from the sociopolitical processes that produce unequal living conditions [1]. Across the U.S., pre‐existing social and health inequities were intensified during the pandemic, leading many local public health officials to seek new strategies for engaging communities and addressing existing social and health inequities.

**FIGURE 1:**
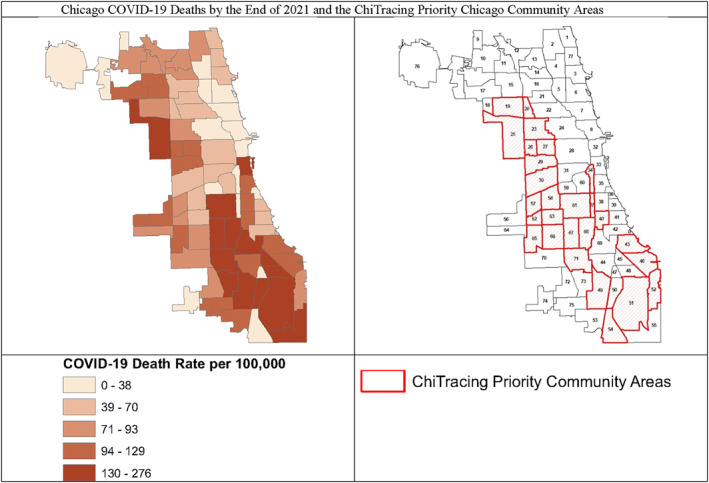
Chicago COVID‐19 deaths by the end of 2021 and the ChiTracing priority Chicago community areas.

Chicago, one of the most racially segregated U.S. cities, has experienced health inequities, even before the pandemic, with a 10+years difference in life expectancy across Chicago's 77 community areas, with predominantly Black neighbourhoods having the lowest life expectancy [2]. COVID‐19 exacerbated such pre‐existing health inequalities. Since the first case was reported on March 4, 2020, Chicago has seen 603,540 reported COVID‐19 cases as of May 20, 2022 (the end of official counting), with a total of 7695 deaths [3]. In spite of shifts in the distribution of COVID‐19 cases and deaths throughout the pandemic, racial inequities persisted. Black and Hispanic residents experienced higher case rates compared to White residents, and COVID‐19 mortality rates were highest on the city's south and west sides, where predominantly Black and Hispanic communities are located (Figure 1).

Spatial segregation by race and class reinforced existing patterns of inequality and produced conditions in which people were differentially exposed to social and environmental risks, while having limited resources to cope with those risks. Early in the pandemic, 70% of COVID‐19 deaths in Chicago occurred among Black residents by June 2020, even though residents who identify as Black comprised only 29.2% of the population [3]. The most affected communities were Burnside, West Englewood, North Lawndale, Pullman, and Roseland, where over 80% of residents identify as Black. South Lawndale, Gage Park, and Hermosa, where over 80% of residents identify as Hispanic, were also disproportionately affected [2]. These predominantly Black and Hispanic communities were vulnerable to social, environmental, and health events, long before COVID‐19.

The health of residents on the west and south sides of Chicago are also significantly affected by social determinants of health such as poverty, high unemployment, low‐wage service jobs, and lack of stable transit; such characteristics put these communities at disproportionate risk of exposure to and adverse outcomes from COVID‐19 infection [2, 4, 5]. Furthermore, high levels of chronic health conditions are concentrated on the west and south sides of Chicago, including diabetes, hypertension, and heart disease, known risk factors for COVID‐19 mortality [6].

Spring 2020, Chicago residents were impacted by significant social events on top of COVID‐19, including the collective recognition of racial injustice brought to national attention through the deaths of George Floyd, Rayshard Brooks, Breonna Taylor, Antonio Valenzuela, Ahmaud Arbery, and others at the hands of police [7].

## 2 Chicago COVID‐19 Response Corps: ChiTracing

In June 2020, the Chicago Department of Public Health (CDPH) collaborated with four partner institutions, including the University of Illinois Chicago School of Public Health (UICSPH), the Sinai Urban Health Institute (SUHI), Malcolm X College (MXC), and NORC at University of Chicago. The Chicago‐Cook Workforce Partnership (The Partnership) coordinated the partner intuitions' activities. Together, this team of organisations formed the Chicago COVID‐19 Contact Tracing Corps (ChiTracing) and was charged with coordinating Chicago's COVID‐19 response. ChiTracing partnered with 31 community‐based organisations (CBOs) from high economic hardship community areas. The CBOs hired and supervised over 460 community residents to work as members of the ChiTracing Corps. ChiTracing was intended to enhance public health infrastructure by investing in a coordinated network of CBOs and developing the Corps of community members who could provide outreach and referrals to hyperlocal resources. The University of Illinois Chicago Institutional Review Board determined this not to be human subjects research.

From its inception, ChiTracing was framed towards an ‘inclusive recovery’ for the city. The approach, grounded in health equity, entailed prioritising those with greatest need, evaluating who benefits and who is burdened by decisions and systems, and engaging people with lived experience that could optimise individual and community level recovery. To realise this vision, ChiTracing identified three primary strategies and related objectives to invest resources into designing and implementing a new **public health infrastructure** by centring trusted local CBOs and people with lived experience, increasing **awareness and knowledge** of public health and available resources for the most vulnerable, and fostering **relationships and power building** among key stakeholders (Table 1).

**TABLE 1:** ChiTracing theory of change (ToC).

Socio‐ecological level on which action impacts		ChiTracing stakeholder group engaged/Impacted		
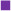 Individual		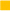 Community residents		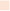 Partner institutions
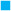 Community		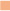 Corps members		
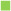 Organisational		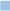 Supervisors		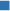 Public health department
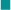 Societal		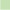 CBO leadership		
Conditions: What are the circumstances in which we do this work	Strategies: How will we do this	Actions: What we do to accomplish impacts	Impacts: What will be different when we are done	Vision
Covid‐19 ◦ Disrupted routine care ◦ Increased interest in public health work Changing norms on who does public health/whose expertise matters Plan for health in Chicago 2025 ◦ Equity commitment/healing opportunities ◦ Health equity work happening in silos History of neglect/broken relationship between communities and city ◦ Community outreach needs to be creative ◦ Inaccurate translations of important information ◦ Inequity in mitigation approaches interventions ◦ Healing opportunity Funding is not expected, certain or streamline (i.e. special funding came out to do this work and newest iteration of work is from multiple funding sources that came together) Work is new and somewhat revolutionary in Chicago	Increase knowledge, awareness: Equip Chicagoans with health knowledge and resources to achieve their desired health outcomes	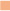 Earn and learn	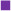 Increase knowledge and confidence of the expanded public health workforce in delivering education and resources	A more responsive, community‐led, public health system in Chicago that relies on hyper local assets and leadership and in which residents are connected to local social determinants of health resources.
		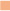 Contact tracing and booster sessions	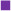 Empowering and supporting people as they navigate their health	
		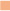 Epidemics of injustice	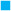 Create community‐level knowledge transfer (about vaccines, housing etc)	
		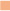 Vaccine ambassadors		
		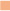 Initiative‐specific training	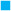 CBOs become trusted source of health information	
	Foster relationships and build power: Increase community members' connections to and utilisation of resources to mitigate the social, economic, and health impacts of the pandemic.	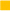 *Promotores de Salud* and street outreach	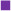 Increase participation in decision making	
		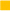 Call centre	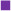 Increase the number of public health advocates	
		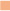 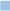 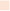 Let's chat	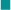 Increase trustworthiness of PH infrastructure	
		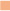 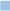 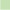 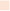 Communities of practice	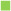 Create and maintain organisational relationships to collaborate/work in solidarity	
		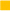 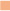 Outreach and events	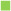 Establish a bridge between community and institutions	
		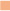 Citizen scientists	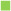 Changing the way that knowledge flows and having communities inform the priorities that get taken up in their community areas	
		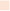 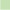 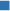 Partner meetings	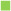 Normalise co‐learning with community members, city, CBOs, the academy	
			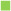 Close the racial life expectancy gap	
	Build public health infrastructure: Continue efforts to promote resilience against COVID‐19 and other public health threats in high‐risk communities while creating a public health workforce comprised of people who live in communities that are most affected by health and economic inequities.	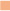 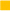 Resource hub	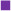 New career pathways for community members with lived experience	
			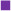 Increased empathy and soft skills of workforce	
			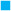 Documenting and integrating hyperlocal narratives of health	
		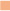 Life scholars	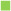 CBOs as advocates of public health	
			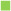 Reinforce trust in public health officials	
		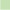 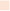 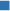 Community centred design institute	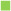 Less siloed public health practice	
			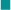 Integration of CBOs into public health infrastructure	
		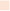 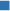 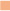 Rapid assessment	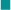 Building a new hyperlocal model of public health	
			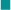 Strategic sustainable funding for public health	
			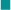 Universal healthcare; transform ambulatory care to be more inclusive	

### 2.1 Infrastructure and Capacity Building

On behalf of CDPH, the Partnership led the process of soliciting, selecting, and contracting of 31 CBOs to serve as local employers of the ChiTracing Corps members. CBOs received assistance with funding application. Applications were scored by the partner institutions on financial stability, service and experience engaging high‐hardship community areas (Figure 1), ability to deliver COVID‐19 prevention education, and CBO's current healthcare and social services provided to the community. The selected CBOs collaboratively launched ChiTracing to increase access to COVID‐19 testing, contract tracing, and wrap‐around social services including food, housing, and financial assistance, and eventually, vaccine outreach. Each CBO recruited and hired 15 community members as the Corps.

ChiTracing also established the Resource Hub (RH) to provide referrals for adjunctive services. The RH deployed resource navigators who assisted individuals and families affected by COVID‐19 with referrals for food or rental assistance. Resource navigators maintained a database of hyperlocal resources via NowPow, a social service platform shared with CBOs and Chicago residents. While the RH was initially for those who had tested positive or were in contact with COVID‐19, the platform was expanded to serve all Chicago residents (Table 2).

**TABLE 2:** ChiTracing programme components.

ToC strategy	Component	Definition
Increase knowledge, awareness	Earn and learn	A programme where ChiTracing corps members were paid to gain skills toward personal career pathways.
	Contact tracing and booster sessions	Initial training designed to teach corps members basic facts about COVID‐19 and contagious disease outbreaks, as well as foundational information about contact tracing
	Epidemics of injustice course	A UICSPH course made available to corps members
	Vaccine ambassadors	Specialised training on COVID‐19, the vaccine, and tactics for addressing community concerns and misinformation.
	Citizen scientists	Specialised training using critical theory and approaches for community‐led research praxis
	Initiative‐specific training	Ongoing on‐the‐job training provided by the partnership on conducting phone calls with scripts, and tips for conducting outreach and canvassing.
Foster relationships and build power	Promotores de salud and street outreach	Efforts to increase street outreach in neighbourhoods and communities hardest hit by COVID‐19
	Call centre work	The corps operated a phone bank that provided contact tracing, vaccination information and scheduling, resource coordination, and health outreach
	Let's chat	Informal conversations with public health leaders and corps members to gain access to critical guidance in a pandemic
	Communities of practice	Shared spaces for co‐learning among corps members and CBO supervisors. These sessions were spaces for didactic learning, relationship building, and sharing feedback/advice.
	Partner meetings	A place for the partners on the effort to provide input and updates. This included CDPH, the partnership, UIC, SUHI, and MXC
Build public health infrastructure	Rapid assessment	A participatory academic‐community collaboration to answer urgent questions about vaccine hesitancy and guide the ChiTracing response
	Resource hub	An effort to connect Chicago residents with social resources, compile and maintain lists of hyperlocal resources.
	Life scholars	Corps members who enroled in degree programs as part of their career goals
	Community centred design institute	A CoP for CBO leaders to strategise a collective impact

### 2.2 Knowledge and Awareness Building

ChiTracing invested in building the Corps members public health knowledge through a variety of job‐specific training programs and interactive, reflexive sessions. Initially, Corps members provided contact tracing and outreach assistance, shifting to vaccine education and distribution. The Partnership with expertise in workforce development, coordinated the expertise of the partner institutions to provide training and ongoing career development. MXC used its expertise in online curriculum and training to develop health‐oriented knowledge and skills. NORC offered expertise in system design and evaluation and the UICSPH provided public health content, evaluation and community‐engaged research expertise. Training protocol included an outreach and canvassing protocol, contact tracing methods, and ‘Earn and Learn’ to expand the knowledge and credentials for public health work beyond the pandemic.

Corps members were paid for training through ‘Earn and Learn’. Topics included: COVID‐19 infectivity and spread, vaccine ambassador training, public health 101, community health worker skills (motivational interviewing and cultural humility), trauma informed outreach, and a Citizen Science training to provide critical theory and approaches for community‐led research praxis. Trainings included informal, discussion‐based co‐learning via Let's Chat sessions and Office Hours with public health experts to address nuanced challenges to outreach due to issues rooted in mistrust.

Trainings provided pathways to community health worker, citizen scientist, community organiser, and higher education, specifically public health degree programs. The Partnership hosted job fairs for Corps members. UICSPH developed the ‘Life Scholars’ programme to assist Corps members interested in higher education to apply to degree programs.

### 2.3 Relationship Building and Power Sharing

SUHI offered grounding on cultural competency and participant engagement via Communities of Practice (CoP) for Corps members and CBO leadership. While didactic training and access to experts were important, CoP provided the time and space for relationship building and co‐learning on best practices and common challenges. CoP sessions were held separately for Corps members, supervisors, and CBO leaders. Sessions on active listening, motivational interviewing, and cultural humility provided a shared language for considering other perspectives. CoP participants asked questions and brainstorm solutions for addressing community concerns or improving their outreach. CBO leaders also shared their expertise on topics such as trauma‐informed outreach to the members of their communities.

The goal of CoPs was to decentralise power so that participants could take ownership over how the Corps operated and benefit from the expertise of their peers. The CBO leadership CoPs were intended to foster networks among CBOs, many of whom had not worked together before, and eventually grew into the Community Centred Design Institute (CCDI) which centred the community expertise in designing new approaches to community outreach and engagement. This CoP developed a theory of change (ToC) that emphasised the importance of advancing health equity by building a more responsive and community‐led public health system in Chicago that relies on hyperlocal assets and leadership.

## 3 Lessons Learnt

Our experience building ChiTracing provides insight into how the three elements of the programme (infrastructure and capacity building, knowledge building and awareness, and relationships and power‐sharing) advanced the overarching goal of health equity. Key lessons emerged across these strategic areas.

First, we learnt that the existing structures and terms of the public health system hindered changes in public health practices. The Public Health Department struggled to quickly staff new public health positions. At the same time, it was difficult for CBO partners, who were often more nimble, to join the public health system because it required seeing alignment of their mission and vision with public health core functions [8, 9]. When asked whether the CBOs had participated in public health initiatives prior to the COVID‐19 pandemic, many said they had not, though their programming was aligned with public health priorities. Nonetheless, an emergent outcome of ChiTracing was the CBOs' expressed desire to continue collaborating and coordinating activities across organisations. And yet, this type of collaboration involving large government funding can risk diverting a CBO's identity and mission. CBOs were provided resources to hire new full‐time employees, whose organisational attention was now focused on ChiTracing not the CBO's central mission. This can lead to the concern that government agencies, like the local health departments, regard CBOs as simply an avenue for reaching constituents. For example, the CBOs became visible as the face of the initiative in their communities and answered for ChiTracing, while not being in control of the direction of the work. In addition, when billing is delayed because of governmental processes, the CBOs must figure out how to maintain operations without compensation straining CBO infrastructure. Further, CBOs carrying out essential public health services for which the government has responsibilities raises questions about neoliberal co‐optation. A hyperlocal public health system that responds to unique community needs while leveraging community assets and resources may be ideal but is a government funded initiative that plays out at the neighbourhood level the same as investing those same resources directly in the neighbourhoods themselves to support a community‐based public health workforce?

Furthermore, all participating CBOs were required to be non‐profit, 501(c)3, organisations with a robust financial infrastructure. These requirements favoured larger and more established CBOs. CDPH addressed this gap by enhancing the RH by tapping into local mutual aid networks that emerged on a local‐level during the pandemic. Mutual aid networks are collective efforts to meet the community's needs that tend to arise in areas where there is a high need that existing governmental resources are not addressing [10, 11]. While the RH led by ChiTracing referred residents to the tangible resources made available by mutual aid networks, none of the ChiTracing funding supported the mutual aid work. Collaborations between state agencies and local mutual aid groups often encounter difficulties in establishing equal partnership because state agencies distribute grant funding with specific goals in mind. Further, collaborations between mutual aid movements and state‐led bureaucracies are often at odds with power sharing, which is fundamental to mutual aid organising. Sustainability of this government‐mutual aid collaboration beyond the pandemic may lead to frictions given contradictions in principles [12].

### 3.1 Scaffolding of Training Leads to Broadened Skills‐Building and Pathways to Higher Education

The ChiTracing Corps members and CBOs received a significant COVID‐19 training. CBOs noted that training in other chronic health conditions would enhance their collective impact by advancing the skillsets and confidence to address community health of those best positioned to serve their communities. This investment of community members to be health ambassadors to their neighbourhoods draws on the important movement to institutionalise community health workers in the public health system [13]. The training programme also resulted in 21 Corps members pursuing a higher education as Life Scholars, most returning to degree programs that had been disrupted due to life stressors.

### 3.2 Importance of Being Responsive to Shifting Needs

Although ChiTracing began as a contact tracing programme, it quickly shifted. The complementary expertise of the partner institutions and CBOs and the rapport developed allowed rapid shifts to new activities. When the vaccine became available, the Let's Chat sessions addressed changes in the project's direction, updated CDC recommendations, and CoPs provided spaces for problem‐solving around outreach.

The introduction of the vaccine led to the division of the CBOs into three specialised activities: outreach work (promotores de salud and street outreach), Call Centre work (vaccine scheduling), and RH connection. Early, when the vaccine was difficult to access, CDPH offered limited blocks of vaccine appointments to residents from high need neighbourhoods, accessed through the Call Centre. CDPH also provided mobile vaccine units, home visits, and telephone‐based scheduling. The CoPs troubleshooted Corps members challenges and generating solutions such as residents' concerns about immigration status and insurance coverage. When the CoP could not solve the issue, SUHI could elevate it to CDPH or it could be raised via a shared platform (Microsoft Teams) that ensured streamlined communication for resolving problems quickly. Developing these bi‐directional communication channels allowed Corps members to impact CDPH directions and decisions.

### 3.3 Centring Equity in a Pandemic

Surveillance is one of the core functions of public health. A major function of CBOs was to enhance COVID‐19 surveillance through contact tracing in order to manage transmission, protect the most vulnerable populations, and inform research, intervention, and policy adjustments. One of the challenges with surveillance is building trust with those affected, so they are willing to share personal information with public health workers. ChiTracing invested in training community members to perform outreach and contact tracing, leveraging the familiarity and shared social context needed to build trust. Inclusion of community members in public health core functions and giving them first‐hand knowledge of accurate and timely data while listening to concerns and interpretations of the data during the pandemic not only centred equity in our public health response but improved the response itself.

From our theory of change we found that building on the knowledge of residents from the most impacted neighbourhoods in the COVID‐19 response, shifting power and building cross‐sectoral relationships and bringing a hyperlocal asset‐based lens was key to maximising our impacts.

## 4 Conclusion

This initiative gave us an opportunity to try something new, and evaluate it: seeing what was effective, what was not, and the effort/money it took to take such an approach. We piloted an inclusive approach grounded in equity and capacity‐building rather than traditional top‐down approaches. Recognising chronic disinvestment in the public health infrastructure which reflects neoliberal capitalist values [14, 15, 16], we sought to invest in a new public health infrastructure by centring trusted CBOs and people with lived experience of systems of oppression as part of the public health system, increasing awareness and knowledge of public health and available resources for the most vulnerable, and fostering relationships and power building among diverse collaborators. We experienced challenges in enhancing the public health workforce with CBOs and Corps members that are rooted in neoliberal capitalism but persevered. Sustaining these collaborations should involve alignment of shared missions and principles of collaborating entities. We were pleased with the ability to scaffold skill‐building among the Corps members and create pathways to the public health workforce, in some cases through higher education. We found our hyperlocal orientation allowed us to quickly be responsive to emergent community needs. We found we were able to centre equity in our multi‐institution, community‐led pandemic response allowing us to respond more quickly to future emergencies. Although, we have been able to learn from this experience to foster equity in other citywide initiatives. We recommend others to implement strategies to build knowledge and awareness of those most impacted by the public health issues, shift power to networked, hyperlocal leadership and prioritise infrastructure that allows for effective communication and equity. The ChiTracing ToC was valuable and could be a roadmap for how to bring a more collaborative, inclusive approach to public health workforce development.

## Data Availability

Data sharing not applicable to this article as no datasets were generated or analysed during the current study.
